# Cardiovascular Magnetic Resonance Reveals Cardiac Inflammation and Fibrosis in Symptomatic Patients with Post-COVID-19 Syndrome: Findings from the INSPIRE-CMR Multicenter Study

**DOI:** 10.3390/jcm13226919

**Published:** 2024-11-17

**Authors:** George Markousis-Mavrogenis, Vasiliki Vartela, Alessia Pepe, Lilia Sierra-Galan, Emmanouil Androulakis, Anna Perazzolo, Aikaterini Christidi, Antonios Belegrinos, Aikaterini Giannakopoulou, Maria Bonou, Agathi-Rosa Vrettou, Fotini Lazarioti, Vasilios Skantzos, Emilio Quaia, Raad Mohiaddin, Sophie I. Mavrogeni

**Affiliations:** 1 University Research Institute of Maternal and Child Health and Precision Medicine and UNESCO Chair in Adolescent Health Care, Medical School, National and Kapodistrian University of Athens, Aghia Sophia Children’s Hospital, 11527 Athens, Greece; georgemm32@gmail.com; 2 Olympic Diagnostic Center, 18537 Piraeus, Greece; faye.lazarioti@gmail.com (F.L.); skantzos.1981@gmail.com (V.S.); 3 Onassis Cardiac Surgery Center, 11527 Athens, Greece; vasvartela@yahoo.gr; 4 Department of Radiology, Medical Faculty, University of Padua, 35127 Padua, Italy; alessia.pepe@ftgm.it (A.P.); anna.perazzolo1@gmail.com (A.P.); emilio.quaia@unipd.it (E.Q.); 5 American British Cowdray Medical Center, Mexico City 05348, Mexico; lilia.sierra@gmail.com; 6 Royal Brompton Hospital, Imaging Centre, Guy’s and St Thomas’ NHS Foundation Trust, London SW3 6NP, UK; em.androulakis@gmail.com (E.A.); r.mohiaddin@rbht.nhs.uk (R.M.); 7 Euromedica MRI Unit, 54124 Thessaloniki, Greece; katechristidi@gmail.com; 8 Faculty of Medicine, National and Kapodistrian University of Athens, 11527 Athens, Greece; a.belegrinos@outlook.com; 9 Aghia Sophia Children Hospital, 11527 Athens, Greece; aikaterinigiannakopoulou@hotmail.com; 10 Laikon Hospital, 11527 Athens, Greece; bonou.maria@yahoo.com; 11 Attikon Hospital, 11527 Athens, Greece; rosa.vrettou@gmail.com; 12 National Heart and Lung Institute, Imperial College London, London SW7 2AZ, UK

**Keywords:** COVID-19, myocarditis, CMR, palpitations, chest pain, shortness of breath

## Abstract

**Introduction**. Post-coronavirus disease-2019 (COVID-19) patients may develop cardiac symptoms. We hypothesized that cardiovascular magnetic resonance (CMR) can assess the background of post-COVID-19 cardiac symptoms using multi-parametric evaluation. We aimed to conduct an investigation of symptomatic patients with post-COVID-19 syndrome using CMR (INSPIRE-CMR). **Methods**. INSIPRE-CMR is a retrospective multicenter study including 174 patients from five centers referred for CMR due to cardiac symptoms. CMR was performed using 3.0 T/1.5 T system (24%/76%, respectively). Myocardial inflammation was determined by the updated Lake Louise criteria. **Results**. Further, 174 patients with median age of 40 years (IQR: 26–54), 72 (41%) were women, and 17 (9.7%) had a history of autoimmune disease, muscular dystrophy, or cancer. In total, 149 (86%) patients were late gadolinium enhanced (LGE)-positive with a non-ischemic pattern, and of those evaluated with the updated Lake Louise criteria, 141/145 (97%) had ≥1 pathologic T1 index. Based on the T2-criterion, 62/173 (36%) patients had ≥1 pathologic T2 index. Collectively, 48/145 (33%) patients had both positive T1- and T2-criterion. A positive T2-criterion or a combination of a positive T1- and T2-criterion were significantly more common amongst patients with severe COVID-19 [45 (31%) vs. 17 (65%), *p* = 0.001 and 32 (27%) vs. 16 (64%), *p* < 0.001, respectively]. During the one-year evaluation, available for 65/174 patients, shortness of breath, chest pain, and arrhythmia were identified in 7 (4%), 15 (8.6%), and 43 (24.7%), respectively. CMR evaluation, available in a minority of them, showed mildly reduced LVEF, while nat T1 mapping and EVC remained at levels higher than the normal values of the local MRI units. **Conclusions**. The majority of post-COVID-19 patients with cardiac symptoms presented non-ischemic LGE and abnormalities in T1 and T2-based indices. Multi-parametric CMR reveals important information on post-COVID-19 patients, supporting its role in short/long-term evaluation.

## 1. Introduction

Convalescent patients with a history of coronavirus disease-2019 (COVID-19) may develop post-COVID-19 cardiac symptoms. The prevalence of cardiac involvement in these patients is unknown, and there is uncertainty as to whether their symptoms correspond to clinical conditions that require treatment. Possible causes include hypoxic injury, stress cardiomyopathy [1,2], ischemic injury caused by microvascular dysfunction, small vessel cardiac vasculitis [3], endotheliitis [4], epicardial coronary artery disease, right heart strain due to pulmonary embolism [5,6,7,8,9], adult respiratory distress syndrome [10], myo-pericarditis [11,12], and systemic inflammatory response syndrome [13,14,15,16].

A recent systematic review showed that myocardial involvement in COVID-19 is associated with a worse prognosis, but isolated myocarditis is not necessarily a marker of poor prognosis. However, due to the small amount of published data and the inhomogeneity of the patients, prognostic conclusions cannot be drawn. Despite the fact that we cannot always demonstrate a cause–effect relationship between SARS-CoV-2 infection and myocarditis, the most recent histology data report direct viral infection of cardiomyocytes [17].

Cardiovascular magnetic resonance (CMR) is the non-invasive gold standard for cardiac function, structure, and tissue characterization. Recent studies identified CMR abnormalities in 26% to 60% of hospitalized patients recovered from COVID-19, including functional impairment, myocardial tissue, or pericardial abnormalities [18]. Patients with mild COVID-19 and asymptomatic individuals are reported to have low rates of CMR abnormalities [18]. In a study of German patients recently recovered from COVID-19 infection, CMR revealed cardiac involvement in 78 patients (78%) and ongoing myocardial inflammation in 60 patients (60%), independent of pre-existing conditions, severity, and overall course of the acute illness and time from the original diagnosis [19]. However, as CMR methods vary significantly among published reports, future studies with standardized CMR protocols are needed to evaluate the long-term effect of COVID-19 disease on the heart [18,19,20].

We hypothesized that CMR can assess the background of post-COVID-19 cardiac symptoms, and we aimed to evaluate the CMR findings in convalescent COVID-19 patients referred due to cardiac symptoms and conducted an investigation of symptomatic patients with post-COVID-19 syndrome using CMR (INSPIRE-CMR).

## 2. Patients-Methods

### 2.1. Patients

INSPIRE-CMR is a retrospective multicenter study including 174 patients from five centers. We included patients referred for CMR from the beginning of the COVID-19 pandemic due to shortness of breath, chest pain, palpitations, or syncope. All patients underwent CMR in a 3.0 T or 1.5 T system (24%/76%, respectively) at a time between 3 and 8 months post COVID-19 infection. All Greek patients gave written inform consent for use of their examination data and the study was approved by the ethics committee of Olympic Diagnostic/Research Center under the protocol code 001, 27 October 2022. Regarding the patients from other countries, they were selected in accordance with the local policy and the declaration of Helsinki.

### 2.2. Methods

The presence of myocardial inflammation was determined using the updated Lake Louise criteria, comprising a T1-criterion [any pathologic T1-index from early/late gadolinium enhancement (EGE/LGE), native T1 mapping, extracellular volume fraction (ECV)] and a T2-criterion (pathologic T2-myocardial to skeletal muscle ratio or T2 mapping) [21,22,23,24,25].

For the T1-criterion, only patients with ≥3/4 measurements available were analyzed (145/174), while for the T2-criterion, any patients with ≥1 index available were analyzed (173/174). Cut-off points for abnormal values (respectively for 1.5 T and 3.0 T) were ≥1000/≥1250 ms for native T1 mapping and >52/>50 for T2 mapping. Pathologic values for ECV, EGE, LGE, and T2-signal ratio were considered for >28%, >4, and >0% of left ventricular mass, and >2, respectively [23,24,25].

### 2.3. CMR Analysis

CMR analysis was performed by two imaging specialists, either a cardiologist or radiologist with at least 5 years of CMR experience, blinded to all identifying information, using commercially available software. LV endocardial and epicardial borders were contoured on the stack of short-axis bSSFP images to assess for left ventricular (LV) volumes, LV ejection fraction (LVEF), and mass, and on short-axis LGE images to assess for the extent of LGE. All LGE images were visually assessed for the presence of LGE. Quantitative LGE analysis was performed using a signal intensity threshold of two standard deviations above a visually normal remote myocardium, expressed as a percentage of total myocardial mass [21,22,23]. The diagnosis of myocarditis was established according to previously published expert consensus papers [21,22].

Native T1, post-contrast T1, and T2 mapping values were evaluated using the mid-ventricular short axis of LV. Regions of interest were covered the whole myocardium, excluding any areas with LGE [23]. Final mapping values were expressed as the mean ± SD of all evaluated short-axis sections. Myocardial extracellular volume (ECV) was calculated with the input of native and post-contrast myocardial and blood pool T1 values, and hematocrit was obtained within 24 h of CMR by non-invasive testing [24,25,26].

### 2.4. Statistical Analysis

Statistical analysis was performed using STATA v14.1 (StataCorp, College Station, TX, USA). A two-tailed *p*-value of <0.05 was considered statistically significant. Continuous variables were described using mean and standard deviation and categorical variables using numbers and percentage. All continuous data were tested for normal distribution using the Shapiro–Wilk test. Comparisons between groups were made using the independent samples t-test for continuous variables with normal distribution, Wilcoxon rank-sum test for continuous variables with non-normal distribution, and Fisher’s exact test for categorical variables. Correlation between variables was assessed using Pearson’s correlation formula.

## 3. Results

In our multicenter COVID-19 convalescent study, patients had a median age of 40 years (IQR: 26–54), 72 (41%) were women, and 17 (9.7%) had a history of autoimmune disease, muscular dystrophy, or cancer. Of 154 with known vaccination status, 116 (75%) were fully vaccinated against SARS-CoV-2, and the majority [148 (85%)] experienced COVID-19 of mild or moderate intensity (Table 1).

**Figure 1:**
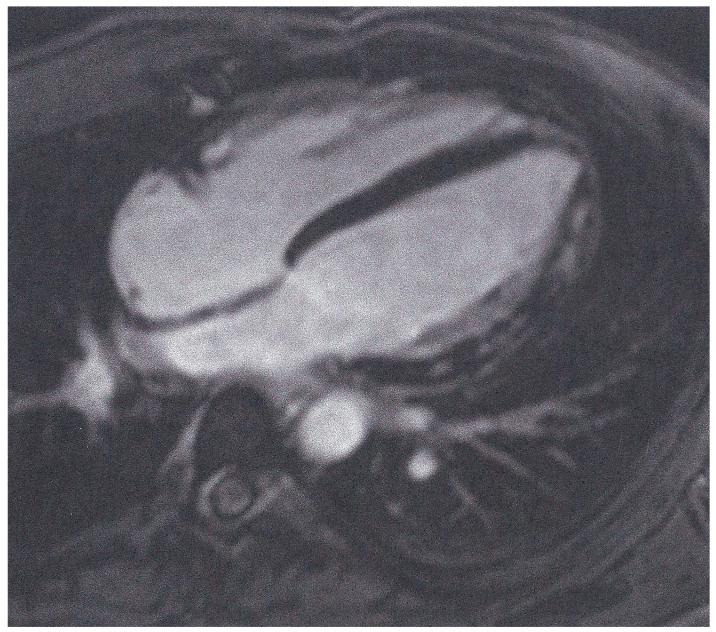
Inversion recovery image of a patient with COVID-19 myocarditis showing LGE in the lateral wall of LV.

**Figure 2:**
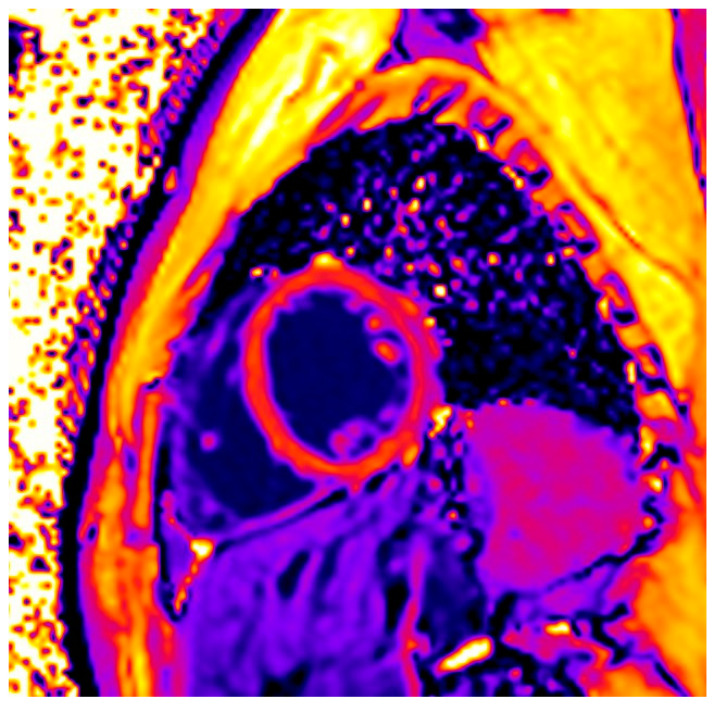
Images presented increased values of nat T1 mapping (1325 msec) due to COVID-19 infection in a patient with myocarditis.

In total, 149 (86%) patients were LGE-positive with a non-ischemic pattern (subepicardial or intramyocardial location) (Figure 1), and of those evaluated with the updated Lake Louise criteria, 141/145 (97%) had ≥1 pathologic T1 index. Based on the T2-criterion, 62/173 (36%) patients had ≥1 pathologic T2 index. Collectively, 48/145 (33%) patients had both a positive T1- and a positive T2-criterion, indicative of high probability of myocardial inflammation (Figure 2). A positive T1-criterion was not associated with a history of severe COVID-19 (*p* = 0.355). However, the presence of a positive T2-criterion or a combination of a positive T1- and T2-criterion were significantly more common amongst patients that had experienced severe COVID-19 [45 (31%) vs. 17 (65%), *p* = 0.001 and 32 (27%) vs. 16 (64%), *p* < 0.001, respectively]. Pericardial enhancement was not identified in any of the examined patients. None of our patients presented evidence of ischemic cardiac disease (subendocardial or transmural LGE pattern) (Table 2).

The one year clinical follow up available in 65/174 patients showed that shortness of breath, chest pain, and arrhythmia were identified in 7 (4%), 15 (8.6%), and 43 (24.7%), respectively. The CMR evaluation, available in a minority of them, showed slightly reduced LVEF, while nat T1 mapping and EVC presented increased values, compared to normal values of the local MRI units.

## 4. Discussion

In our multicenter COVID-19 convalescent study, we found that CMR identified the presence of non-ischemic LGE in the majority of patients. Furthermore, it revealed abnormalities in T1-based indices in most patients and s high probability of myocardial inflammation in ~1/3 of patients with history of COVID-19 and cardiac symptoms. A history of severe COVID-19 was associated with a higher probability of abnormalities in T2-based indices alone and/or in combination with abnormal T1-based indices. The one-year clinical follow up, available in 65/174 patients, showed shortness of breath, chest pain, and arrhythmia were identified in 4%, 8.6%, and 24.7%, respectively. CMR evaluation available in a minority of them showed slightly reduced LVEF, and increased values of nat T1 mapping and EVC.

An international online survey study of 3762 patients found that cardiac symptoms including chest pain (∼53%), palpitations (∼68%), and fainting (∼13%) were observed in up to ∼86% of patients by 7 months from infection [27]. Furthermore, another study that assessed the prevalence of long COVID among 2550 patients using a social media survey found that cardiopulmonary symptoms were reported by 89% of participants in their study [28]. Our findings regarding the prevalence of cardiac symptoms in post-COVID-19 patients were in agreement with these studies.

The American College of Cardiology, the European Society of Cardiology, and the Society for Cardiovascular Magnetic Resonance recommended CMR as a valuable tool in patients with COVID-19 presenting with myocardial injury and evidence of cardiac dysfunction [29,30,31,32]. CMR has already been used in post-COVID-19 patients. A prospective study of 100 recovered patients showed that the majority (49%) of them had mild-to-moderate COVID-19 and 2/3 of them were not hospitalized. At 2 to 3 months after a positive test result, 78 of 100 patients with prior COVID-19 had an abnormal CMR finding [19]. Furthermore, the mean LV and RV ejection fractions were lower and the median native T_1_ and T_2_ were higher (indicative of edema and/or collagen deposition) than in control individuals. Pericardial enhancement was frequent (22%). There were greater proportions of patients with ischemic (32% vs. 17%) and nonischemic (20% vs. 7%) LGE patterns than the risk factor-matched control group. However, individuals not hospitalized for COVID-19 had fewer CMR abnormalities compared to the hospitalized patients [19]. In another study including 26 patients recovered from COVID-19, cardiac involvement was found in 58% of them and CMR identified myocardial edema, fibrosis, and impaired right ventricular function [33]. Lastly, a recent systematic review showed that the rate of raised T1 in COVID-19 adult survivors varied across studies from 0% to 73%. Raised T2 was not detected in patients in 4 out of 15 studies, and in the remaining studies, its rate ranged from 2% to 60%. In most studies, LGE (myocardial or pericardial) was observed in COVID-19 survivors, with the rate ranging from 4% to 100%. Myocardial LGE had mainly nonischemic patterns. Most studies found that patients who recovered from COVID-19 had a significantly greater T1 and T2 compared to participants in the corresponding control group [34].

The findings of our multicenter study were in agreement with the results of the systematic review. Furthermore, 1/3 of our patients had both a positive T1- and a positive T2-criterion indicative of a high probability of myocardial inflammation. Additionally, while a positive T1-criterion was not associated with a history of severe COVID-19, the presence of a positive T2-criterion or a combination of a positive T1- and T2-criterion were significantly more common amongst patients with severe COVID-19. Our findings were in agreement with previous studies supporting that both native T1 and T2 values are increased, and that they can therefore be used as indicators of active myocardial inflammation [35]. However, native T1 was less strong compared to T2 in the detection of acute myocardial inflammation. This is expected, as it was proven in experimental animals with inflammatory heart disease that myocardial inflammation, evaluated histologically, peaked at day 21 and decreased by day 35, but remained still higher than baseline values from day 0 [36]. Our findings are in agreement with this experimental study, as the majority of our patients were evaluated at least after three months of COVID-19, and T1 mapping represents an index of very early inflammatory reaction. This may explain the lack of correlation between native T1 mapping and COVID-19 severity, while a positive T2-criterion and/or a combination of a positive T1- and T2-criterion were significantly more common in patients who experienced severe COVID-19. Furthermore, the CMR evaluation at least 3 months post COVID-19 infection had potentially led to lack of pericardial enhancement in our patients.

CMR, based on the detection of tissue edema, hyperemia, and necrosis, was proven to be a valuable non-invasive diagnostic tool for the assessment of myocarditis. However, the classic Lake Louise Criteria (LLC) had decreased sensitivity for the diagnosis of chronic myocarditis (greater than 8 weeks post symptoms onset). Emerging sequences such as T_1_ and T_2_ parametric mappings provide tissue characterization regarding inflammation without reliance on reference tissue and thus overcome the limitations of the LLC [37]. These indices have proven useful not only in diagnosis but also in the characterization of the diseased tissue, prognostication, and clinical decision making [37]. Furthermore, according to the recent literature, T1 mapping and application of the modified LLC provided important diagnostic value, with T1 mapping providing prognostic value in patients with Immune Checkpoint Inhibitor-Associated (ICI) myocarditis [38].

Our patients can be clinically characterized as having chronic myocarditis, as all of them had cardiac symptoms with consequent CMR evaluation at a time between 3 and 8 months from COVID-19 and the onset of cardiac symptoms. An interesting finding in our patients is that the 1/3 of them still had increased T2 mapping and/or a combination of increased native T1 and T2 mapping values. This supports that COVID-19 can still provoke an acute myocardial reaction months after COVID-19 infection. Taking into consideration that the patients with increased native T1 and T2 mapping had a history of more severe COVID-19 disease, we assume that CMR multi-parametric imaging may not only lead to diagnosis, but also to a better understanding of COVID-19’s effect on the myocardium and potentially better prognostication of these patients [37]. Furthermore, the CMR findings have important clinical implications, as they can provide details about the extent and acuity of myocardial involvement, the risk for future cardiac events, and may potentially motivate the application of endomyocardial biopsy (EMB) in cases where it is needed [37].

Another important finding of our study is the presence of an exclusive non-ischemic LGE pattern in our patients. This is in agreement with the results of a systematic review involving 1601 articles that also identified the non-ischemic LGE pattern [34]. Lastly, our finding supporting that the development of myocardial inflammation was independent of the severity of COVID-19 infection is also in agreement with a previous study [19].

To our knowledge, our study represents the first multicenter evaluation of COVID-19 patients with cardiac symptoms using CMR. Our results reassessed the already known results of the systematic review [34]. The main finding was a pattern of non-ischemic myocardial inflammatory disease. However, acute pericardial involvement was not identified, probably because the CMR evaluation was performed at least 3 months after COVID-19 infection.

There are only scarce studies presenting one-year follow up of COVID-19 patients. In one of these, CMR abnormalities including LV/RV dysfunction and/or abnormal T1mapping occurred in one in five individuals with long COVID-19 at 6 months and persisted in over half of those at 12 months [39]. In our study, the one-year clinical follow up available in around 1/3 of patients identified the presence of shortness of breath, chest pain, and arrhythmia. The CMR follow up available in a minority of them showed slightly reduced LVEF, while nat T1 mapping and EVC presented increased values, compared to the normal population. Our results are in agreement with this study, supporting the role of CMR in short/long-term evaluation of COVID-19 patients with cardiac symptoms. Additionally, we have to mention that in the context of arrhythmias, some studies show that the number of real arrhythmias experienced by the patients was not increased after undergoing COVID-19, as they are considered as sinus tachycardia and not real arrhythmias [40]. However, we should note that sinus tachycardia is the most common clinical sign of myocardial inflammation [41].

Despite the high incidence of CMR lesions post COVID-19, we still do not know their clinical impact in the development of future cardiac events and, therefore, further multicenter studies with long-term follow up are needed to identify their role in the development of heart failure and sudden cardiac death. However, observational data from other viral myocarditis studies suggests the important role of CMR, with LGE being the strongest independent predictor of SCD, cardiac, and all-cause mortality. Of them, patients who develop HF and arrhythmias usually have a larger LGE involving several myocardial segments. Nevertheless, there is no formal consensus about the extension of LGE to justify implantable cardioverter defibrillator (ICD) implantation in primary prevention [42]. Finally, another study comparing COVID-19 myocarditis with other non-COVID-19 viral myocarditis using CMR and EMB showed that both CMR and EMB found that SARS-CoV-2 infection was associated with relatively mild but variable cardiac involvement. Of those with COVID-19 myocarditis, those with more symptomatic COVID-19 disease were more likely to develop chronic inflammation and impaired cardiac function compared to those with milder forms of the disease [43]. Lastly, we should always keep in mind that SARS-CoV-2 infection may result in persistent effects on the body vasculature, leading to long-term COVID-19, characterized by prolonged inflammation, endothelial dysfunction, and a high risk of vascular complications, which can be diagnosed using various imaging modalities and histopathologic evaluation [43,44].

## 5. Limitations

The most important limitations of our study include the following:

(1) The retrospective design of our study may have led to selection biases of patients referred for CMR. This may have resulted in an under- or over-estimation of patients with cardiac involvement.
(2) The lack of 1-year CMR re-evaluation in the majority of the patients.
(3) The lack of >1-year follow up of the patients.

The main cause for the lack of long-term CMR evaluation is that the treating physicians consider CMR as a tool for the recent evaluation of myocarditis, but they are still not aware of its clinical value for the long-term evaluation of these patients. Low availability and high cost are other additive causes. The lack of long-term CMR re-evaluation leads to underutilization of this powerful modality and consequently the loss of valuable information that may potentially influence therapeutic approaches.

The results of our multicenter study strongly support the need for the CMR evaluation of all patients recovered from COVID-19 infection who present with cardiac symptoms, irrespective of COVID-19 severity. However, further studies are needed to evaluate the consequences of cardiac involvement post COVID-19 disease in cardiac morbidity/mortality and the long-term survival of these patients.

## 6. Conclusions

In convalescent post-COVID-19 patients with cardiac symptoms, CMR revealed abnormalities in T1-based indices in most patients, and a high probability of myocardial inflammation in ~1/3 patients, irrespective of the severity of COVID-19 disease. Severe COVID-19 infection was associated with a higher probability of abnormalities in T2-based indices alone or in combination with abnormal T1-based indices. The high incidence of active myocardial inflammation long-term post COVID-19 infection strongly supports the need for multi-parametric CMR for the evaluation of these patients. Lastly, the 1-year clinical follow up identified shortness of breath, chest pain, and arrhythmia. Furthermore, the CMR re-evaluation, available in a minority of patients, showed slightly reduced LVEF, while native T1 mapping and ECV remained above the normal limits. These findings further support the significant role of CMR in the short/long-term follow up of these patients. However, despite the high percentage of CMR lesions, we still do not know their clinical impact in the development of future cardiac events.

## Figures and Tables

**Table 1:** Demographic and clinical characteristics of COVID-19 patients included in INSPIRE study.

Population Characteristics	COVID-19 Patients Characteristics (n = 174)
Age	26–54 yrs
Sex	72 (41%) women
Autoimmune disease, muscular dystrophy, cancer	17 (9, 7%)
Known fully vaccinated pts	116 (75%)
Mild or moderate intensity of COVID-19 disease	148 (85%)

**Table 2:** CMR characteristics of COVID-19 patients included in INSPIRE study.

CMR Parameters	CMR Findings in COVID-19 Patients (n = 174)
Non-ischemic LGE	149 (86%)
Ischemic LGE	None
≥1 pathologic T1 index	141/145 (97%)
≥1 pathologic T2 index	62/173 (36%)
Both positive T1- and T2-criterion	48/145 (33%)
Pericardial enhancement	None

## Data Availability

The data that support the findings of this study are available from the corresponding author upon reasonable request.
